# Bailey and Bishop's Notable Names in Medicine and Surgery

**Published:** 1984-04

**Authors:** J. D. Davies

**Affiliations:** Department of Pathology University of Bristol


### Book Review

**BAILEY AND BISHOP'S NOTABLE NAMES IN MEDICINE AND SURGERY**

**Revised by H. Ellis**

**H. K. Lewis & Co. Ltd., London, 4th Edition, 1983**

**272 pp.,+xv, hardback, Price £15.00**

There seems to be renewed interest in short biographies on the part of many publishing houses. General biographies are available in paperback,[1] surprisingly on the booksellers' remainder shelves,[2] and in hardback[3] *Serious Scientists'* lives are also available. Little wonder that the *British Medical Journal* recently has been debating the possible virtues of Do-it-Yourself obituaries.

To tell the truth, until two months ago I had never even seen *Bailey and Bishop's Notable Names in Medicine and Surgery*, which is now forty years old and in its Fourth Edition. Since arriving in our house the book has been in continual use; even non-medical members of the family have disappeared with it. No, it's not pornographic. Oddly the book has a distinctly Victorian flavour and unfashionably is not at all anti-heroic. In this sense the book is not a product of of our times, and even lacks the epigrammatic venom of John Aubrey's seventeenth-century biographies.

We all know the embarrassing silence in examination halls that may follow the question, 'Tell me, who was . . . Langerhans, Lugol or Lister?' Fresh Candidate and weary Examiner all too often fail together in their attempt at polite conversation. Soon they are both likely to be back to debating the dogma of current biomedical mythology. How much more pleasant if part of the time could be passed in discussion of whether a marble bust on the staircase to the *locus* of the *Vice Voce* was justifiable. In this book lies diversion from the most wearisome of vivas. Grab the chance: the next question is almost certainly, 'Tell me, why are Thromboxane, Testicular Teratomas or Tapeworms of importance today?'

For sensible souls not contemplating examination encounters this book offers wider philosophical enquiry. Hamilton Bailey's own life style is clearly reflected in the number of workaholics who are included. Nonetheless Vincent (and his angina) and Corrigan (the pulse) achieved immortality with remarkably short lists of publications. Neither longevity nor marital stability seem necessary for those bent on fame. Unexpected curiosities pop out all over the place. Can you imagine how they administer the general Cook County Hospital in Chicago with its 3400 beds? Did you know that University College Hospital was called the North London Hospital in the 1880's? Were you aware that most of Gram's work was as a physician? John Cheyne (1777–1836, of Cheyne-Stokes' respiration) seems unlikely to have successfully completed an UCCA form, let alone to have become a professor. Even publication in the 'right' journal seems not to have been necessary to enter Bailey and Bishop's select company. So much for network theory, and the incredibly expensive Science Citation Index and its games.

Not only are the stories there, but the text is liberally sprinkled with illustrations, even if many are rather too small after the publisher's reductions. It is all good fun. Criticisms? Well, obviously even to the amateur historian there are doubts. On the other hand did such factual reservations ever spoil the pleasure for the reader of Boyd's *Textbook of Pathology*? Part fiction or pure fact: does it matter in an introduction? Perhaps Harold Nicolson was correct in writing: 'Biography is the preoccupation and the solace, not of certainty but of doubt.'[4] We all thrive on such possibilities. Myth and truth are inseparable. The main deficiency in the book is that there are not yet more lives included. To the serious minded a set of brief bibliographic references and suggestions indicating further reading are appended. For the tyro and your reviewer, this book is an entertaining introduction to the delights of medical biography. Purchase is thoroughly recommended. Otherwise, like me, you are likely to be deprived by the continual absence of the book on any library shelf.

J. D. Davies
Department of Pathology
University of Bristol

**REFERENCES**

1. BULLOCK, A. and WOODINGS, R. B. (1983) The Fontana biographical companion to modern thought. London, Fontana Paperbacks.
2. WINTLE, J. (1981) Makers of modern culture. London, Routledge & Kegan Paul.
3. WILLIAMS, T. I. (1982) A biographical dictionary of scientists. 3rd Edition. London, Adam & Charles Black.
4. NICHOLSON, H. (1927) The development of English biography. London, The Hogarth Press. p. 65.

